# Diagnostic utility of salivary LDH and MMP-9 in differentiating oral cancer from potentially malignant disorders

**DOI:** 10.6026/973206300220230

**Published:** 2026-01-31

**Authors:** Ragini Motwani, Yash Saxena, Ipsita Sharma, Maheshwari Shanmugam, Nidhi Dhakar, Swati SP, Tanvi Hirani

**Affiliations:** 1 Department of Oral Pathology and Microbiology, Peoples Dental Academy, Bhopal, Madhya Pradesh, India; 2 Department of Pathology, Government Medical College, Satna, Madhya Pradesh, India; 3 Department of Oral Pathology & Microbiology, RKDF Dental College & Research Centre, Bhopal, Madhya Pradesh, India; 4 Department of Oral Pathology and Microbiology, JKKN Dental College and Hospital, Komarapalayam, India; 5 Department of Oral Pathology and Oral Microbiology, Government Dental College and Hospital, Ahmedabad, Gujarat, India; 6 Department of Public Health Dentistry, MGM Dental College & Hospital, Kamothe, Maharashtra, India; 7 Department of Periodontology and Implantology, Karnavati School of Dentistry, Karnavati University Gandhinagar, Gujarat, India

**Keywords:** Oral squamous cell carcinoma, potentially malignant disorders, salivary biomarkers, lactate dehydrogenase, matrix metalloproteinase-9, early diagnosis, non-invasive diagnostics

## Abstract

Early distinction between oral squamous cell carcinoma (OSCC) and possibly malignant disorders (PMDs) has been one of the most important diagnostic issues that may involve invasive biopsies. Therefore, it is of interest to assess the usefulness of salivary lactate dehydrogenase (LDH) and matrix metalloproteinase-9 (MMP-9) as non-invasive biomarkers of early detection. Spectrophotometry and ELISA were used in analyzing saliva samples of 135 participants (50 OSCC, 45 PMDs, 40 controls). The level of LDH and MMP-9 was much greater in OSCC than in PMDs and controls and demonstrated very high diagnostic accuracy (AUC > 0.89). Simultaneous biomarker measurement had better sensitivity and specificity indicating their possible use as well-grounded, non-invasive measures of screening oral cancer in its early stages.

## Background:

Oral squamous cell carcinoma (OSCC) is the most common malignancy of the oral cavity with an incidence rate of about 90 per cent of all oral malignancies and is a major global health issue with an estimated 377,000 new cases each year worldwide [1]. Although therapeutic modalities have improved, the five-year survival rate of OSCC is very dismal at about 50-60 years; this is mainly due to late diagnosis at full stages when the treatment modalities are minimal and prognosis is severely affected [2]. Early diagnosis and treatment is still the most important factor that determines positive clinical outcomes and survival. Potentially malignant disorders (PMDs) of the oral cavity, such as oral leukoplakia, erythroplakia, oral submucous fibrosis and oral lichen planus are heterogeneous diseases having different rates of risk of malignant transformation [3]. Malignant transformation rate of PMDs vary between 0.13 to 34 percent based on the disorder in question, clinical presentation and dysplasia [4]. Correct diagnosis and risk classification of PMDs and dependable distinction between current malignancies must be undertaken in order to manage them properly, surveillance guidelines and treatment. At present, the gold standard of OSCC diagnosis and the dysplastic alteration of PMDs is still the histopathological examination of the tissue biopsy [5]. Nevertheless, traditional biopsy-based diagnosis has some fundamental drawbacks such as invasiveness, discomfort to the patient, sampling error in heterogeneous lesions, inter-observer variation in histological reading and inability to carry out longitudinal follow-up of multi or extensive lesions in practice. These limitations have raised the interest of doing research in order to develop non-invasive, dependable and affordable diagnostic methods using biological fluids, which are easily available.

Saliva has already become an attractive diagnostic medium, commonly referred to as the mirror of the body and has many benefits such as non-invasive collection, easy repeated sampling, sufficient volume supply and locally and systemically derived molecular markers, which indicate physiological and pathological conditions [6]. The idea of salivary diagnostics has become highly momentum when many cancer associated biomarkers such as proteins, enzymes, nucleic acids and metabolites are identified [7]. Lactate dehydrogenase (LDH) is a ubiquitous cytoplasmic enzyme that plays a role in the interconversion of pyruvate and lactate molecules in the glycolysis pathway and has been widely studied as a cancer biomarker [8]. Higher LDH levels of the biological fluids indicate higher cellular turnover, tissue destruction and also the metabolic alteration toward glycolysis modes of the malignant cells (Warburg effect). In the past, it has been observed that salivary LDH activity is high in patients with OSCC relative to normal people [9]. Nevertheless, extensive data comparing the level of LDH between the spectrums of health to PMDs to developed malignancy are scarce. The gelatinase B, or also called matrix metalloproteinase-9 (MMP-9), is a member of a group of zinc-dependent endopeptidases that perform the degradation of the extracellular matrix (ECM) [10]. MMP-9 has central roles in invasion of tumors, metastasis, angiogenesis and immune modulation. MMP-9 has been shown to be overexpressed in malignancies, such as OSCC, where it promotes degradation of the basement membranes, migration of tumor cells and tumor cell metastasis [11]. Several studies have indicated the Salivary MMP-9 increase in oral cancer patients, but its discriminatory ability between PMDs and OSCC needs to be clarified [12]. Although there is some early positive evidence, the current literature still has a number of significant gaps. To begin with, the majority of studies have made comparisons of the level of biomarkers between OSCC patients and healthy controls, but little research has been done on the clinically vital difference between PMDs and early malignancy. Second, the diagnostic performance (sensitivity, specificity and area under curve) properties of these biomarkers in terms of differentiating between them have not been done thoroughly. Third, the possibility of a synergistic effect of biomarker combination in improving diagnostic accuracy should be explored. Fourth, biomarkers that are evaluated should be correlated with the clinicopathological parameters and disease evolution in a systematic fashion [13]. Therefore, it is of interest to evaluate salivary LDH and MMP-9 levels across healthy individuals, PMDs and OSCC patients, assessing their individual and combined diagnostic performance (sensitivity, specificity, AUC) for distinguishing PMDs from early malignancy, while correlating with clinicopathological parameters to address existing literature gaps.

## Materials and Methods:

## Study design and ethical approval:

This hospital-based cross-sectional observational study was conducted at the Department of Oral Medicine and Radiology in collaboration with the Department of Biochemistry between June 2023 and August 2024.

## Sample size calculation:

Sample size was calculated using MedCalc Statistical Software based on the primary objective of comparing biomarker levels among three groups. Assuming an effect size of 0.40, alpha error of 0.05 and power of 85% for detecting significant differences using one-way ANOVA, a minimum of 38 participants per group was required. Accounting for potential sample inadequacy or dropout (approximately 15%), we aimed to recruit 45 participants per group.

## Study population and selection criteria:

A total of 135 participants were enrolled and categorized into three groups:

Group 1 (OSCC group, n=50): Patients with histopathologically confirmed primary OSCC of the oral cavity who had not received any prior treatment (surgery, chemotherapy, or radiotherapy).

Group 2 (PMD group, n=45): Patients clinically and histopathologically diagnosed with oral potentially malignant disorders including leukoplakia (n=22), erythroplakia (n=8) and oral submucous fibrosis (n=15).

Group 3 (Control group, n=40): Age- and gender-matched healthy volunteers with no clinical evidence of oral mucosal lesions, systemic diseases, or habits predisposing to oral cancer.

## Inclusion criteria for OSCC and PMD groups:

(1) Age 25-75 years; (2) histopathologically confirmed diagnosis; (3) treatment-naïve status; (4) ability to provide adequate saliva sample (≥2 mL); (5) willingness to participate with informed consent.

## Inclusion criteria for control group:

(1) Age and gender-matched to study groups; (2) absence of oral mucosal lesions; (3) no history of malignancy; (4) systemically healthy.

## Exclusion criteria (all groups):

(1) History of previous malignancy or current malignancy at other sites; (2) recent oral surgical procedures within 4 weeks; (3) active oral infections (bacterial, viral, or fungal); (4) severe periodontal disease; (5) autoimmune disorders or immunosuppressive therapy; (6) pregnancy or lactation; (7) current use of antibiotics, anti-inflammatory drugs, or immunomodulatory medications; (8) smoking or tobacco cessation less than 3 months; (9) salivary gland disorders; (10) recent dental prophylaxis within 2 weeks.

## Clinical investigation and data gathering:

Thorough clinical assessment was done by two qualified oral medicine investigators. Standardized proforma was used to record demographic data, medical history, tobacco consumption and alcohol consumption and clinical characteristics. The lesion site, lesion size, clinical involvement and staging (in case of OSCC) were recorded. Each OSCC case was staged based on the TNM classification system (8 th edition, American Joint Committee on Cancer).

## Saliva collection protocol:

Saliva samples had been taken within the range of 9:00 to 11:00 AM to ensure that circadian variation was kept to a minimum. The participants were requested to: (1) avoid eating, drinking, smoking, or oral hygiene procedure at least 2 hours before collection; (2) rinse mouth with much water 10 minutes before collection. The unstimulated whole saliva (passive drool method) was measured by requesting the participants to fill the mouth with saliva and spit into sterile graduated collection tubes within a 10-minutes time. The collected volume was a minimum of 2 mL. Samples were taken to the laboratory on ice.

## Salivary processing and storage:

Centrifugation of saliva samples was done at 3000 rpm in 15 minutes at 4°C to remove cellular debris and particulate matter. Micro pipets were then used to collect clear supernatant and aliquot it in sterile cryovials. Samples were instantly frozen and kept at -80°C until bio-chemical analysis. The analysis of all samples was done within a period of 3 months of collection. Single use aliquots were used, preventing freeze-thaw cycles.

## Biochemical analysis:

Salivary LDH Activity Measurement: The activity of LDH was measured by commercially available kinetic spectrophotometric assay kit (Randox Laboratories Ltd., UK) on the basis of conversion of lactate to pyruvate and the reduction of the NAD + into NADH at 340 nm wavelength. Semi-automatic biochemistry analyzer (Erba Chem-5 Plus) was used to analyze it. The results were in units per liter (U/L). Measurements were done in duplicate and mean values obtained. The intra-assay coefficient of variance was less than 5%. Salivary MMP-9 Concentration Measurement: MMP-9 concentration was evaluated by commercially available sandwich enzyme-linked immunosorbent assay (ELISA) kit (R & D Systems, Minneapolis, USA) according to the instructions provided by the manufacturer. In a nutshell, 100 0.1 ml of diluted saliva samples and normal saliva standards were put in microplate wells that were pre-treated with monoclonal antibody against MMP-9. The incubation, washing and adding of enzyme-conjugated detection antibody and substrate solution was followed by optical density at 450 nm reading with microplate reader (Bio-Rad Model 680). The standard curve was determined and the concentration was calculated as nanograms per milliliter (ng/mL) of MMP-9. Every sample was tested in two samples. Intra-assays and inter-assays coefficient of variation were less than 6 and 8 percent respectively. The quality control was done through positive and negative controls, calibration standards and blinding of lab personnel to groups.

## Histopathological examination:

All OSCC and PMD patients were subjected to incisional or excisional biopsies after the collection of saliva. The specimens were fixed by using 10% neutral buffered formalin, processed and embedded in paraffin, sectioned to a thickness of 4-5 0.00mm thickness and stained with hematoxylin and eosin. Two blinded oral pathologists that did not know the biomarker outcomes were used to perform the histopathological evaluation. Cases of OSCC were classified according to well-differentiated, moderately differentiated and poorly differentiated. PMD cases were evaluated in terms of the presence and the degree of epithelial dysplasia (none dysplasia, mild, moderate and severe dysplasia).

## Statistical analysis:

The SPSS Statistics version 26.0 (IBM Corporation, USA) and MedCalc Statistical Software version 20.0 were used to analyze statistics. The continuity variables were tested on the normality by Shapiro-Wilk test and reported as mean + standard deviation or median (interquartile range) respectively. Ultimately, categorical variables were provided in the form of frequencies and percentages. To compare the levels of biomarkers in three groups, one-way analysis of variance (ANOVA) was applied, along with post-hoc post-test (Tukey). Two group comparisons were done by using independent t-test. Pearson or Spearman calculated correlation coefficients were used to evaluate the relationship between biomarkers and clinicopathological variables. In order to define the diagnostic performance, to define the optimal cut-off values (Youden index) and to estimate the area under curve (AUC), sensitivity and specificity, positive and negative predictive values (PPV and NPV), receiver operating characteristic (ROC) curve analysis was conducted. Combined biomarker assessment was done using logistic regression analysis. The level of statistical significance was set to p<0.05.

## Results:

A total of 135 participants were enrolled in the study, comprising 50 OSCC patients, 45 PMD patients and 40 healthy controls. No significant differences existed among groups regarding age and gender distribution, ensuring adequate matching (Table 1). Presents demographic and clinical characteristics of the study population. The mean age was comparable across groups (p=0.267). Gender distribution showed male predominance in all groups without significant differences (p=0.518). As expected, tobacco consumption and alcohol use were significantly higher in PMD and OSCC groups compared to controls (p<0.001). Among OSCC patients, 64.0% presented with advanced stage (III-IV) disease and buccal mucosa was the most common site (42.0%). Both salivary LDH activity and MMP-9 concentration demonstrated progressive elevation from controls through PMDs to OSCC, with statistically significant differences among all groups (Table 2).

Mean salivary LDH levels were 182.3 ± 44.8 U/L in controls, 318.6 ± 74.2 U/L in PMD patients (74.7% increase from controls) and 580.4 ± 118.7 U/L in OSCC patients (218.3% increase from controls, 82.2% increase from PMDs). All pairwise comparisons were statistically significant (p<0.001). Similarly, mean salivary MMP-9 concentrations were 96.7 ± 28.4 ng/mL in controls, 186.4 ± 51.2 ng/mL in PMD patients (92.8% increase) and 422.8 ± 93.5 ng/mL in OSCC patients (337.2% increase from controls, 126.8% increase from PMDs), with all differences being statistically significant (p<0.001). In PMD patients, both LDH and MMP-9 levels showed significant positive correlation with degree of dysplasia (LDH: r=0.68, p<0.001; MMP-9: r=0.72, p<0.001). Patients with moderate-to-severe dysplasia had significantly higher biomarker levels compared to those with no/mild dysplasia (LDH: 389.4 ± 62.5 vs 278.2 ± 54.8 U/L, p<0.001; MMP-9: 234.6 ± 48.3 vs 157.8 ± 38.6 ng/mL, p<0.001). In OSCC patients, both biomarkers demonstrated significant associations with clinical stage and histological grade. Advanced stage (III-IV) tumors exhibited higher levels than early stage (I-II) tumors (LDH: 618.7 ± 108.4 vs 512.3 ± 98.6 U/L, p=0.002; MMP-9: 458.3 ± 86.7 vs 362.4 ± 77.2 ng/mL, p<0.001). Poorly differentiated tumors showed elevated biomarkers compared to well-differentiated tumors (LDH: 672.1 ± 115.3 vs 532.8 ± 102.4 U/L, p=0.008; MMP-9: 501.4 ± 94.8 vs 378.6 ± 82.3 ng/mL, p=0.003). ROC curve analysis was performed to evaluate the diagnostic utility of salivary LDH and MMP-9 for differentiating OSCC from PMDs, which represents the clinically most relevant discrimination (Table 3). Salivary LDH demonstrated excellent discriminatory ability with AUC of 0.892 (95% CI: 0.819-0.945, p<0.001). Using optimal cut-off value of 425.0 U/L, sensitivity was 84.0% and specificity was 82.2% for differentiating OSCC from PMDs. Salivary MMP-9 showed superior performance with AUC of 0.918 (95% CI: 0.852-0.962, p<0.001). At optimal cut-off of 278.0 ng/mL, sensitivity was 88.0% and specificity was 86.7%. Combined biomarker analysis using logistic regression model yielded the highest diagnostic accuracy with AUC of 0.953 (95% CI: 0.895-0.985, p<0.001), sensitivity of 92.0% and specificity of 88.9%. The combined approach demonstrated significantly better performance than either biomarker alone (p=0.024 for LDH comparison; p=0.041 for MMP-9 comparison using DeLong test). For differentiating OSCC from healthy controls, both biomarkers showed even higher diagnostic performance (LDH: AUC 0.975, sensitivity 96.0%, specificity 92.5%; MMP-9: AUC 0.984, sensitivity 96.0%, specificity 95.0%).

## Discussion:

The diagnostic value of salivary LDH and MMP-9 as non-invasive biomarkers to distinguish between oral squamous cell carcinoma and potentially malignant conditions and healthy controls is shown. The key findings show that the two biomarkers are considerably increased in a progressive stage between healthy people to PMDs to proven OSCC with good diagnostic performance features. The integrated analysis of biomarkers also increased the diagnostic accuracy, which is supportive to the possible clinical use of salivary biomarkers in the screening and risk stratification of oral cancer. Salivary LDH activity of the OSCC patients (580.4 U/L) is higher than salivary activity of PMDs (318.6 U/L) and control (182.3 U/L) and it is in line with other previous studies and it can be explained by numerous pathophysiological processes [14]. The characteristics of malignant transformation include metabolic reprogramming, namely, the Warburg effect, when the metabolism of cancer cells preferentially follows glycolytic metabolism even in aerobic conditions which leads to higher levels of LDH expression and activity [15]. Also, increased cellular turnover, necrosis and permeability of the membrane of the quickly proliferating tumor cells contribute to high levels of LDH discharge into the surrounding tissues and biological fluids. The gradual increase of the controls to the PMDs then OSCC indicates that the LDH levels can be used as an indicator of the malignant transformation continuum which can be used to indicate the progression of the disease. Salivary LDH sensitivity and specificity of Shetty *et al.* were found to be 83.3% and 76.7 per cent respectively as far as oral cancer detection was concerned [14]. Nonetheless, our research goes further and methodically assesses the median PMD group and determines certain threshold values of differentiating diagnosis. The relationship between the LDH level and the tumor stage and grade that is observed in this disease further add to its use as a disease severity indicator, which is in agreement with the serum LDH trends in other malignancies [8].

High concentrations of salivary MMP-9 in OSCC (422.8 ng/mL) relative to PMDs (186.4 ng/mL) and controls (96.7 ng/mL) are indicative of the importance of the role of matrix metalloproteinases in cancer invasion and metastasis [16]. MMP-9 enhances the breakdown of type IV collagen in the basement membranes and the extracellular matrix, which allows invasion of tumor cells, angiogenesis via released trapped growth factors and metastatic spread [17]. Malignant epithelial cells and tumor-associated stromal cells such as fibroblasts, inflammatory cells and endothelial cells produce the enzyme, which helps in the tumor microenvironment that facilitates progression. Manek *et al.* also reported significantly elevated salivary MMP-9 in OSCC patients vs. controls (p<0.05), with higher levels in poorly differentiated tumors, confirming MMP-9's role in tumor microenvironment progression [18]. In OSCC, Salivary MMP-9 levels were also associated with tumor invasiveness as well as lymph node metastasis [19]. These findings are supported by our study which sheds more light on the MMP-9 elevation in PMDs, especially dysplastic ones. The substantial association between the level of MMP-9 and the grade of dysplasia implies that the process of matrix remodeling may be initiated in the multistep carcinogenesis early before the appearance of the histologically important malignancy. Clinically, this finding of intermediate levels of both the biomarkers in PMD patients especially those with moderate-severe dysplasia has significant implications. This gradation is indicative that these markers could be used to risk stratify the PMD category and detect lesions that have greater risk of malignant transformation and which should be subjected to more intensive surveillance or earlier intervention. Such non-invasive monitoring of biomarker trends over time may supplement histopathological assessment, having a sampling bias and incapable of being repeatedly done at frequent intervals.

The value of multi-markers in cancer diagnostics can be highlighted by the fact that the combination of biomarker analysis (AUC 0.953, sensitivity 92.0, specificity 88.9) outperforms single biomarkers. LDH and MMP-9 provide information that is complementary to each other, whether it is biology of cancer, metabolic dysregulation or invasive potential, respectively and their combination give better information of the disease [20]. Such a multi-marker approach is in line with the current concept of precision medicine and the understanding that cancer is a heterogeneous disease that needs a multifaceted method of diagnosis [21]. This study has a number of strengths that are worth mentioning. To begin with, having a well-defined PMD group is a necessary clinical requirement of biomarkers capable of distinguishing between premalignant and malignant diseases. Second, extensive correlation with clinicopathological measurements such as TNM stage, histological grade and severity of dysplasia gives information on biomarker-disease associations. Third, by using strict sample collection procedures, standardized methods of analysis, quality control procedures and blind assessment, bias is minimized. Fourth, ROC curve analysis and optimal cut-off values give diagnostic thresholds, which are immediately applicable. There are however some shortcomings that should be mentioned. The cross- sectional design does not allow determining longitudinal changes of biomarkers of disease progress or response to treatment. The sample size is relatively small (especially with respect to the subgroup analysis (initially, PMD types, tumor stages) and this can be a limitation of the generalization. The sample size of one tertiary care institution might not be reflective of the overall prevalence and disease range of the community. Potential confounding variables such as tobacco consumption which influences disease risk and salivary composition should be considered with a lot of care. The absence of validation in an independent cohort provides the need to interpret it with caution until external validation is done.

## Conclusion:

Salivary LDH and MMP-9 are promising non-invasive valid biomarkers to be used in the early diagnosis and differentiation of oral squamous cell carcinoma and potentially malignant conditions. Their joint evaluation improves the accuracy of diagnostic value. It is associated with the disease severity which can be used in screening and monitoring. Additional multicentric, large-scale validation is needed for standard oral cancer detection.

## Figures and Tables

**Table 1:** Demographic and clinicopathological characteristics of study participants

**Characteristic**	**Control Group (n=40)**	**PMD Group (n=45)**	**OSCC Group (n=50)**	**p-value**
Age (years), mean±SD	48.2±11.4	50.3±12.8	52.6±13.2	0.267
Gender, n (%)				0.518
Male	24 (60.0%)	29 (64.4%)	34 (68.0%)	
Female	16 (40.0%)	16 (35.6%)	16 (32.0%)	
Tobacco habits, n (%)	0 (0%)	38 (84.4%)	46 (92.0%)	<0.001*
Alcohol consumption, n (%)	0 (0%)	22 (48.9%)	35 (70.0%)	<0.001*
**PMD type, n (%)**				
Leukoplakia	-	22 (48.9%)	-	
Erythroplakia	-	8 (17.8%)	-	
Oral submucous fibrosis	-	15 (33.3%)	-	
**Dysplasia grade in PMD, n (%)**				
No dysplasia	-	12 (26.7%)	-	
Mild dysplasia	-	18 (40.0%)	-	
Moderate dysplasia	-	11 (24.4%)	-	
Severe dysplasia	-	4 (8.9%)	-	
**OSCC tumor site, n (%)**				
Buccal mucosa	-	-	21 (42.0%)	
Tongue	-	-	15 (30.0%)	
Alveolar ridge	-	-	8 (16.0%)	
Others	-	-	6 (12.0%)	
**OSCC clinical stage, n (%)**				
Stage I-II	-	-	18 (36.0%)	
Stage III-IV	-	-	32 (64.0%)	
**OSCC histological grade, n (%)**				
Well-differentiated	-	-	24 (48.0%)	
Moderately differentiated	-	-	19 (38.0%)	
Poorly differentiated	-	-	7 (14.0%)	
PMD = Potentially malignant disorders;
OSCC = Oral squamous cell carcinoma;
*Statistically significant (p<0.05)

**Table 2:** Comparison of salivary LDH and MMP-9 levels among study groups

**Biomarker**	**Control Group (n=40)**	**PMD Group (n=45)**	**OSCC Group (n=50)**	**p-value (ANOVA)**
**Salivary LDH (U/L)**				
Mean±SD	182.3±44.8^a^	318.6±74.2^b^	580.4±118.7^b^	<0.001*
Median (IQR)	178.5 (150.0-210.0)	312.0 (268.0-365.0)	572.0 (495.0-658.0)	
Range	98.0-285.0	185.0-485.0	345.0-845.0	
**Salivary MMP-9 (ng/mL)**				
Mean±SD	96.7±28.4^a^	186.4±51.2^b^	422.8±93.5^b^	<0.001*
Median (IQR)	92.5 (76.0-115.0)	182.0 (148.0-220.0)	415.0 (352.0-485.0)	
Range	45.0-165.0	95.0-298.0	235.0-648.0	
**Post-hoc comparisons (p-values)**				
LDH: Control vs PMD	-	<0.001*	-	
LDH: Control vs OSCC	-	-	<0.001*	
LDH: PMD vs OSCC	-	-	<0.001*	
MMP-9: Control vs PMD	-	<0.001*	-	
MMP-9: Control vs OSCC	-	-	<0.001*	
MMP-9: PMD vs OSCC	-	-	<0.001*	
LDH = Lactate dehydrogenase;
MMP-9 = Matrix metalloproteinase-9;
IQR = Interquartile range;
Different superscript letters (a, b, c)
indicate statistically significant
differences among groups (Tukey's post-hoc test);
*Statistically significant (p<0.05)

**Table 3:** Diagnostic performance of salivary biomarkers for differentiating OSCC from PMDs

**Parameter**	**Salivary LDH**	**Salivary MMP-9**	**Combined Biomarkers**
Area under curve (AUC)	0.892	0.918	0.953
95% Confidence interval	0.819-0.945	0.852-0.962	0.895-0.985
p-value	<0.001	<0.001	<0.001
Optimal cut-off value	425.0 U/L	278.0 ng/mL	-
Sensitivity (%)	84.00%	88.00%	92.00%
Specificity (%)	82.20%	86.70%	88.90%
Positive predictive value (%)	84.00%	88.00%	90.20%
Negative predictive value (%)	82.20%	86.70%	90.90%
Accuracy (%)	83.20%	87.40%	90.50%
Positive likelihood ratio	4.72	6.62	8.28
Negative likelihood ratio	0.19	0.14	0.09
Diagnostic odds ratio	24.84	47.29	92
LDH = Lactate dehydrogenase;
MMP-9 = Matrix metalloproteinase-9;
Combined biomarkers assessed using
logistic regression model
